# Interleukin-22 Inhibits Bleomycin-Induced Pulmonary Fibrosis

**DOI:** 10.1155/2013/209179

**Published:** 2013-02-18

**Authors:** Minrui Liang, Jiucun Wang, Haiyan Chu, Xiaoxia Zhu, Hang He, Qiong Liu, Jianhua Qiu, Xiaodong Zhou, Ming Guan, Yu Xue, Xiangjun Chen, Hejian Zou

**Affiliations:** ^1^Division of Rheumatology, Huashan Hospital, Fudan University, 12 Wulumuqi Zhong Road, Shanghai 200040, China; ^2^Institute of Rheumatology, Immunology and Allergy, Fudan University, Shanghai 200040, China; ^3^Ministry of Education Key Laboratory of Contemporary Anthropology and State Key Laboratory of Genetic Engineering, School of Life Sciences, Fudan University, Shanghai 200433, China; ^4^Department of Pancreatic Surgery, Pancreatic Disease Institute, Huashan Hospital, Fudan University, Shanghai 200040, China; ^5^Department of Human Anatomy, Histology and Embryology, Shanghai Medical College, Fudan University, Shanghai 200032, China; ^6^Department of Neurology and Radiology, Massachusetts General Hospital, Harvard Medical School, Charlestown, MA 02129, USA; ^7^Division of Rheumatology and Clinical Immunogenetics, Department of Internal Medicine, The University of Texas Medical School, Houston, TX 77030, USA; ^8^Department of Clinical Laboratory, Huashan Hospital, Fudan University, Shanghai 200040, China; ^9^Department of Neurology, Huashan Hospital, Fudan University, Shanghai 200040, China

## Abstract

Pulmonary fibrosis is a progressive and fatal fibrotic disease of the lungs with unclear etiology. Recent insight has suggested that early injury/inflammation of alveolar epithelial cells could lead to dysregulation of tissue repair driven by multiple cytokines. Although dysregulation of interleukin- (IL-) 22 is involved in various pulmonary pathophysiological processes, the role of IL-22 in fibrotic lung diseases is still unclear and needs to be further addressed. Here we investigated the effect of IL-22 on alveolar epithelial cells in the bleomycin- (BLM-) induced pulmonary fibrosis. BLM-treated mice showed significantly decreased level of IL-22 in the lung. IL-22 produced **γ** **δ**T cells were also decreased significantly both in the tissues of lungs and spleens. Administration of recombinant human IL-22 to alveolar epithelial cell line A549 cells ameliorated epithelial to mesenchymal transition (EMT) and partially reversed the impaired cell viability induced by BLM. Furthermore, blockage of IL-22 deteriorated pulmonary fibrosis, with elevated EMT marker (**α**-smooth muscle actin (**α**-SMA)) and overactivated Smad2. Our results indicate that IL-22 may play a protective role in the development of BLM-induced pulmonary fibrosis and may suggest IL-22 as a novel immunotherapy tool in treating pulmonary fibrosis.

## 1. Introduction

Pulmonary fibrosis can occur as an idiopathic disease or as a consequence of a variety of connective tissue diseases with undefined aetiology, including scleroderma, dermatomyositis/polymyositis, systemic lupus erythematosus, and rheumatoid arthritis. Pulmonary fibrosis is characterized by epithelial injury and activation, formation of distinctive subepithelial fibroblast/myofibroblast foci, and excessive extracellular matrix (ECM) accumulation. Many lines of evidence have suggested that recurrent injuries to pulmonary epithelial cells and ineffective repair initiate aberrant fibroblastic responses. Epithelial cells undergo phenotypic changes of epithelial to mesenchymal transition (EMT), in which the cells lose their epithelial characteristics and acquire a mesenchymal phenotype. It is estimated that up to one third of fibroblasts may be of epithelial origin according to lineage tracing in murine models of lung fibrosis *in vivo* [1, 2]. Although this view has been challenged by Rock et al. [3], it needs further study not only in mice but also in tissues from patients with idiopathic pulmonary fibrosis. The key mesenchymal features of pathological fibrosis are increased numbers of transdifferentiated fibroblasts, named myofibroblasts. These cells share features with both fibroblasts and smooth muscle cells. They overexpress *α*-smooth muscle actin (*α*-SMA) and are probably responsible for the enhanced synthesis of abnormal matrix observed in pulmonary fibrosis [4]. Transforming growth factor (TGF)-*β*1 has been shown to play a key role in pulmonary fibrosis, not only through its functions to attract fibroblasts and to stimulate their proliferation, but also through induction of EMT in alveolar epithelial cells by activating Smad- or non-Smad signaling pathways [5].

Interleukin-22 (IL-22) is a member of the IL-10 cytokine family and plays a critical role in inflammation, immune surveillance, and homeostasis in tissues that serve a barrier function such as skin, respiratory (trachea and lungs) and gastrointestinal (stomach, small intestines, and colon) tracts as well as liver, pancreas, and kidney [6]. IL-22 is produced by special T and natural killer (NK) cell subsets. Cells of nonhematopoietic origin express the IL-22 receptor and respond to it. IL-22 binds to a membrane receptor complex composed of the IL-22R1 (IL-22RA1) and IL-10R2 (IL-10RB2) [7], and signals intracellularly mainly through transcription factor JAK/STAT [8]. 

Increasing evidence suggested that IL-22 is associated with respiratory damages. Interestingly, it was also showed that TGF-*β*/Smad signaling contributes to BLM-induced fibrosis by promoting EMT, and recent studies demonstrated that TGF-*β* downregulated the IL-22 producing capacity of Th17 cells in both human [9, 10] and mouse systems [11] and inhibited the development of Th22 cells [12]. Similarly, IL-17A could regulate the properties of IL-22 in the airway damage and inflammation [13], whereas IL-17A enhanced BLM-induced fibrosis in a TGF-*β* dependent manner [14]. To date, however, the crosstalk between IL-22 and TGF-*β*-driven EMT in pulmonary fibrosis has remained unclear.

In the present study, we investigated the role of IL-22 in EMT in BLM-induced pulmonary fibrosis mouse model as well as *in vitro*. We found that IL-22 inhibited BLM-induced EMT, suggesting a potential therapeutic role of IL-22 in pulmonary fibrosis.

## 2. Materials and Methods

### 2.1. Bleomycin-Induced Pulmonary Fibrosis Mouse Model

C57BL/6 mice were purchased from the Shanghai Laboratory Animal Center (Chinese Academy of Sciences). The animal study was approved by the institutional animal care and use committee of Huashan Hospital, Fudan University. All surgery was performed under chloral hydrate anesthesia, and all efforts were made to minimize suffering. Six- to eight-week old female C57BL/6 mice were used for the studies of pulmonary fibrosis. For BLM-induced pulmonary fibrosis, mice were anaesthetized with 2% chloral hydrate and administered BLM (Nippon Kayaku) intratracheally at a dose (uL) of 3.5 units/kg dissolving in total 50 ul saline. Control groups were injected with 50 uL saline in the same fashion. Mice were sacrificed at weeks 1, 3, 6, and 8 after BLM injection. Bronchoalveolar lavage fluid (BALF) was collected. The left lungs were fixed in 10% formalin, dehydrated, and embedded in paraffin. The right lungs were frozen in liquid nitrogen for the subsequent protein and mRNA experiments.

For the *in vivo* experiment, mice were divided into 4 groups at random: the first and second group were given BLM as described above and injected intraperitoneally with 1.25 *μ*g anti-IL-22 neutralizing monoclonal antibody (Ab) or isotype Ab (both from PerproTech) suspended in saline for 2 consecutive weeks, respectively; the third and fourth group were just given once BLM or saline, respectively, through intratracheal route, serving as BLM control and saline control.

### 2.2. Cell Culture and Stimulation

Human type II alveolar epithelial cells (A549) were a gift from Jiucun Wang's lab (obtained from the American Type Culture Collection, ATCC). A549 cells were harvested in F-12 K medium containing 10% fetal bovine serum (FBS) with 100 U/mL penicillin and 100 ug/mL streptomycin at 37°C in a humidified 5% CO_2_ atmosphere. Confluent cultures of A549 were serum-starved for 12 hours (h) and then cultured with or without 100 mU/mL BLM, subsequently stimulated with recombinant human IL-22 (PerproTech) of different concentrations for 48 h. Cell viability was measured by cell counting kit-8 (CCK-8, Dojindo).

### 2.3. Flow Cytometry for Intracellular Staining

After sterile phosphate buffered saline (PBS) was infused through the pulmonary vasculature by right heart puncture to remove any contaminating peripheral blood mononuclear cells, the whole lung was digested with collagenase IV (GIBCO) and DNase I (Sigma) at 37°C for 60 minutes (min) on the shaker. After filtering, erythrocyte lysing, and two washes with PBS, mononuclear cells from lung homogenates were incubated in 24-well plates with RPMI 1640 medium containing 10% FBS. For intracellular cytokine staining, total lung cells were cultured at 10^6^ cells/mL in complete RPMI 1640 medium containing cell stimulation cocktail (plus protein transport inhibitors) (eBioscience, including phorbol 12-myristate 13-acetate (PMA), ionomycin, and protein transport inhibitors—brefeldin A and monensin) at 37°C for 5 h. The cells were washed and stained with monoclonal antibodies directed against CD3 (APC, eBioscience), CD4 (FITC, eBioscience), *γδ*TCR (FITC, eBioscience), or NKp46 (FITC, eBioscience). Cells were fixed and permeabilized with flow cytometry staining buffer (ebioscience) and permeabilization buffer (ebioscience) per manufacturer's instructions, followed by staining with IL-22 (PE, eBioscience), or IL-17A (PE, eBioscience), or isotype controls for 30 min at room temperature. The lymphocyte population was identified using forward and 90° light scatter patterns, and fluorescence intensity was analyzed using a FACS Canto cytometer (BD).

### 2.4. Real-Time Reverse Transcriptase-Polymerase Chain Reaction (RT-PCR) Assay

Total RNA was isolated from frozen lung specimens using TRIZOL Reagent (invitrogen) in accordance with the manufacturer's protocols. PrimeScript RT reagent Kit (TaKaRa) was used to reverse-transcribe 1 ug RNA to complementary DNA. Real-time RT-PCR (40 cycles of denaturation at 95°C for 15 seconds and annealing at 60°C for 60 seconds) was performed on an ABI Prism 7500 sequence detector (Applied Biosystems) with SYBR Premix Ex Taq (TaKaRa). GAPDH was used to normalize the mRNA level. The relative expressions of PCR products were determined according to the ΔΔC_t_ method which compares target gene and GAPDH messenger RNA (mRNA) expression. 

### 2.5. Western Blot

Total protein concentration was measured using the BCA protein assay kit (Biyuntian) with bovine serum albumin (BSA, Sigma-Aldrich) as the standard protein. Thirty *μ*g of protein were loaded for each lane of 10% SDS PAGE gels, followed by electrophoresis, and protein transfers to PVDF membranes (Millipore). After the transfer, membranes were blocked with 5% BSA. Immunoblots were probed with primary antibody against STAT3 (Cell Signaling Technology), pSTAT3 (Cell Signaling Technology), *α*-SMA (Epitomics), E-cadherin (Bio-world), IL-22 (Millipore), Smad2 (Cell Signaling Technology), pSmad2 (Cell Signaling Technology), or GADPH (Epitomics) at 4°C overnight followed by goat anti-rabbit secondary antibodies (Jackson ImmunoResearch, 1 : 10,000 dilution) for 30 min at room temperature. After extensive washing, the immunoblots were visualized by ECL (Pierce) and the band densities for each phenotype marker were quantified using Image Reader Las-3000 (Fijifilm) after scanning with a Las-3000 Imaging Densitometer (Fujifilm).

### 2.6. Histological Analysis

Paraffin-embedded sections (4 *μ*m in thickness) were stained with hematoxylin and eosin (H&E) and Masson's trichrome (Sigma-Aldrich) according to the manufacturer's instructions. For immunohistochemistry analysis, sections were treated with 3% hydrogen peroxide for 10 minutes at room temperature to block endogenous peroxidase. Subsequently, the sections were incubated with antibody against IL-22 (Millipore), *α*-SMA (Epitomics), TGF-*β* (Santa Cruz), Collagen I (Abcam), or Collagen III (Abcam) overnight at 37°C, and then incubated at 37°C for 1 hour with horseradish peroxidase-conjugated goat anti-rabbit IgG secondary antibody. Sections were washed 3 times with PBS between each incubation. After development with 3,3′-diaminobenzidine tetrahydrochloride and hydrogen peroxide, sections were counterstained with hematoxylin.

### 2.7. Statistics

Values were expressed as the mean ± SD. An independent two-group *t*-test or one-way analysis of variance (ANOVA) test with LSD's multiple comparison test was used to evaluate the significance of differences between groups. *P* values less than 0.05 were considered statistically significant.

## 3. Results

### 3.1. Pulmonary Fibrosis Induced by Intratracheal Injections of BLM Undergoes EMT

**Figure 1:**
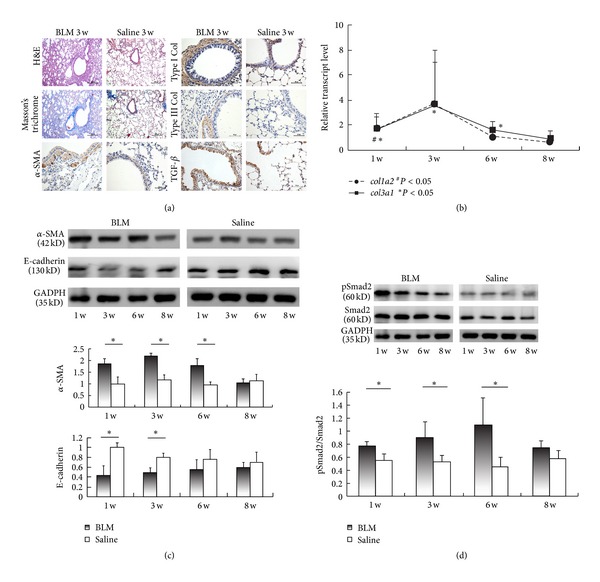
Bleomycin-treated mice undergo robust epithelial to mesenchymal transition (EMT). (a) Representative photomicrographs of lung sections were obtained from mice treated with bleomycin (BLM) or saline (week 3). Sections were stained with hematoxylin and eosin (H&E), and Masson's trichrome, as well as by immunohistochemistry for anti-*α*-smooth muscle actin(*α*-SMA), anti-collagen I (Col I), anti-collagen III (Col III), anti-transforming growth factor (TGF)-*β* antibody. Bars = 200 *μ*m (for H&E and Masson's trichrome staining), or 50 *μ*m (for immunohistochemistry). (b) Expressions of *col1a2* and *col3a1* from the lung tissues of BLM-treated mice were measured by real-time reverse transcription-polymerase chain reaction (RT-PCR) analysis relative to that of saline-treated mice.  ^#^ *P* < 0.05 (for *col1a2*); *P < 0.05 (for *col3a1*). (c) Protein levels of *α*-SMA, E-cadherin and GADPH in lung homogenates were determined by western blotting and analyzed by densitometry compared to GADPH expression. The same amounts of total protein are loaded in each lane. (d) Protein levels of phosphorylated Smad2 (pSmad2), total Smad2, and GADPH in lung homogenates were determined by western blotting and analyzed by densitometry compared to GADPH expression. The same amounts of total protein are loaded in each lane. Fold increase in pSmad2 expression after normalization to total Smad2 expression is shown. * = *P* < 0.05; ** = *P* < 0.01. Data presented in (b), (c), and (d) are representative of results from two independent experiments with five mice in each group. Values are shown as mean ± SEM.

To determine if the EMT response was involved in pulmonary fibrosis after BLM administration, we examined pathological changes and EMT markers (E-cadherin for epithelial cells and *α*-SMA for myofibroblasts) in the lungs over an 8-week period. In this study, pulmonary fibrosis in C57BL/6 mice was examined by H&E, Masson's trichrome, immunohistochemistry for collagen (Col) I and Col III staining at the 1st, 3rd, 6th, and 8th week after BLM or saline treatment, showing that the treatment with BLM enhanced the collagen deposition in the lung tissues (Figure 1(a)). Apart from smooth muscle cells of vascular and bronchiolar walls, *α*-SMA was mainly expressed in myofibroblast (Figure 1(a)). It was shown that *α*-SMA was upregulated, whereas E-cadherin was downregulated in BLM-treated mice (Figure 1(c)). The increased transcripts of *col1a2* and *col3a1* (Figure 1(b)) and protein level of *α*-SMA (Figure 1(c)) showed that the lung fibrosis aggravated after BLM treatment and peaked around the 3rd week. These data indicated that BLM-induced lung fibrosis is characterized by an EMT response.

TGF-*β* expression in the lung tissues of BLM-treated mice was higher than that of saline-treated mice (Figure 1(a)). Meanwhile, both phosphorylated (pSmad2) and total Smad2 in the lung tissues of BLM-treated mice were found to be elevated (Figure 1(c)), and the ratio of pSmad2/Smad2 was significantly increased at the 1st, 3rd, and 6th week (Figure 1(d)). These results suggested that the TGF-*β*/Smad2 related EMT process participated in the initiation and development of the pulmonary fibrosis.

### 3.2. Decreased Expression of IL-22 in BLM-Induced Pulmonary Fibrosis 

**Figure 2:**
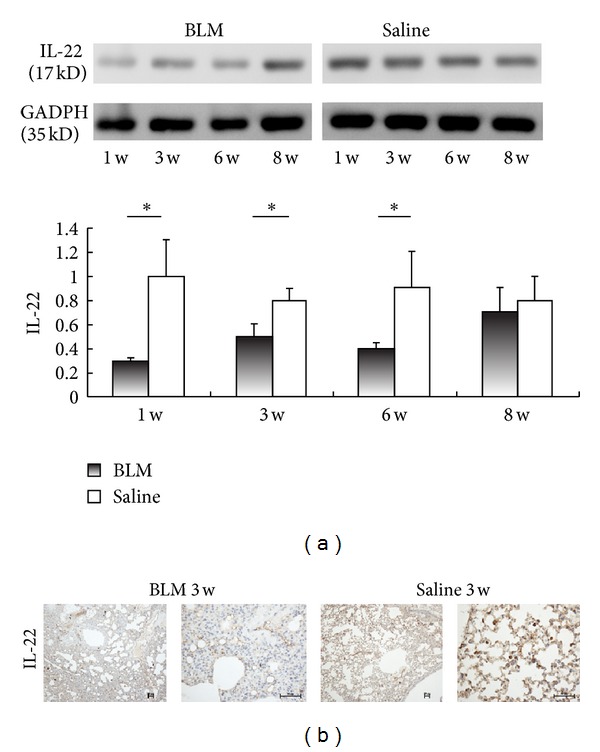
IL-22 is downregulated in lungs from bleomycin-treated mice. (a) Total protein level of IL-22 in lung homogenates was determined by western blotting and analyzed by densitometry compared to GADPH expression. The same amounts of total protein are loaded in each lane. Experiments were performed twice with similar results, with the same amounts of total protein loaded in each lane. (b) Representative photomicrographs of lung sections were obtained from mice treated with BLM or saline (week 3). IL-22-positive cells were examined by immunohistochemistry. Bars = 50 *μ*m. * = *P* < 0.05; ** = *P* < 0.01. Data presented in (a) are representative of results from two independent experiments with five mice in each group. Values are shown as mean ± SEM.

To determine whether IL-22 was involved in BLM-induced pulmonary fibrosis, the expression of IL-22 was evaluated by western blotting (Figure 2(a)), or immunohistochemistry (Figure 2(b)). As shown by immunoblots, total IL-22 production in the lung tissue was significantly decreased in the BLM-treated mice during 8-week period (Figure 2(a)), which was in agreement with the histological findings (Figure 2(b)). In addition, most IL-22-positive cells were showed to distribute mainly subepithelially, within the alveoli and vessels. Decrement of IL-22 level, either secreted or *in situ*, implicated a potential role of IL-22 in pulmonary fibrosis.

### 3.3. Differential Expression of IL-22 and IL-17A by CD4^+^T, TCR*γδ* ^+^T, NKp46^+^ Cells in BLM-Induced Pulmonary Fibrosis

**Figure 3:**
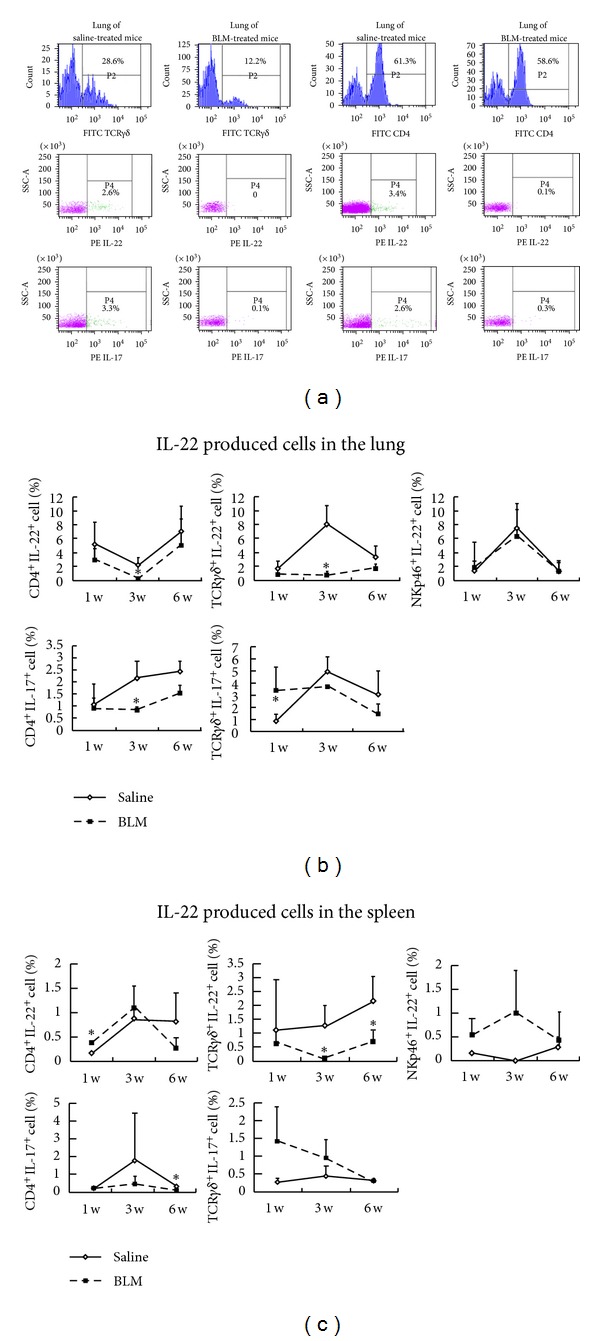
Infiltration of IL-22/IL-17 produced cells in the lungs and spleens induced by intratracheal injections of bleomycin (BLM). Representative flow cytometry pictures of CD4^+^IL-22^+^ and TCR*γδ* ^+^IL-22^+^ cell from the lung and spleen tissues at the 3rd week were shown in Figure 1(a). And the percentages of CD4^+^IL-22^+^, TCR*γδ* ^+^IL-22^+^, NKp46^+^IL-22^+^, CD4^+^IL-17^+^, and TCR*γδ* ^+^IL-17^+^ cells in the lungs (b) and spleens (c) were counted by flow cytometry analysis at the indicated week (w). Data presented in (b) and (c) are from two independent experiments with 8~10 mice in each group. Values are the mean and SEM. *P < 0.05.

To better understand the origin of IL-22 and IL-17 in BLM-induced pulmonary fibrosis, the percentages of IL-22 and IL-17 produced cells were examined in the lung and spleen tissues of C57BL/6 mice after BLM treatment by flow cytometry. In the lung tissues, as compared with saline-treated mice, the percentages of CD4^+^IL-22^+^, TCR*γδ* ^+^IL-22^+^, and CD4^+^IL-17^+^ cells were significantly reduced in BLM-treated mice, especially at the 3rd week after the treatment (Figure 3(a)). In contrast, BLM-treated mice showed significantly increased percentage of TCR*γδ* ^+^IL-17A^+^ T cells at the 1st week in the lung tissues of BLM-treated mice. In the spleen tissues, the percentages of TCR*γδ* ^+^IL-22^+^, NKp46^+^IL-22^+^, and CD4^+^IL-17^+^ cells were reduced, but CD4^+^IL-22^+^ and TCR*γδ* ^+^IL-17A^+^ were increased at the 1st week (Figure 3(b)). In addition, very few IL-17A^+^NKp46^+^ cells were found in the lung and spleen tissues within the same period (data not shown). These data indicated that CD4^+^ and TCR*γδ* ^+^ T cells differentially expressed IL-17A and IL-22 in response to BLM treatment, suggesting that the subsets of CD4^+^IL-22^+^, TCR*γδ* ^+^IL-22^+^, CD4^+^IL-17A^+^, and TCR*γδ* ^+^IL-17A^+^ T cells may have distinct functions in BLM-induced pulmonary fibrosis.

### 3.4. Amelioration of BLM-Induced EMT of Alveolar Epithelial Cell A549 by IL-22

**Figure 4:**
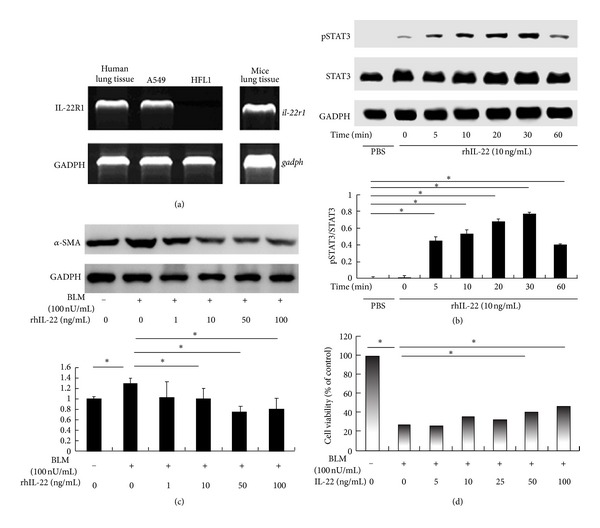
IL-22 attenuates bleomycin-induced epithelial mesenchymal transition (EMT) and impaired cell viability of alveolar epithelial cells. (a) Reverse transcription-polymerase chain reaction (RT-PCR) analysis was performed for IL-22R1 and GADPH on total RNA isolated from the whole lungs of human and mice, as well as human cell lines A549 and HFL1. PCR products were analyzed in ethidium bromide-2% agarose gels. (b) Human alveolar epithelial cell line A549 was treated with 10 ng/mL IL-22 at the indicated length of time. Levels of phosphorylated STAT3, total STAT3, and GADPH were determined by western blotting and analyzed by densitometry compared to GADPH expression. Fold increase in pSTAT3 expression after normalization to total STAT3 expression is shown. The experiments were performed thrice with similar results. (c) and (d) After induction by bleomycin (BLM) (100 mU/mL), A549 was treated with or without IL-22 at the indicated concentrations for 48 h. Protein levels of *α*-smooth muscle actin (*α*-SMA) and GADPH were determined by western blotting in cell homogenates and analyzed by densitometry compared to GADPH expression (c). Cell viability was examined by cell counting kit-8 (CCK-8) analysis (d). Each condition included 6~8 wells. *P < 0.05, **P < 0.01. Results in (c) represent one out of three independently performed experiments with similar outcomes. Values in (b), (c), and (d) are shown as mean ± SEM. *P < 0.05; **P < 0.01.

To further explore the underlying mechanism of IL-22 in BLM-induced pulmonary fibrosis, we examined IL-22 expression in the lung. Studies have shown that IL-22R1, a specific receptor, was mainly expressed in primary alveolar epithelial cell (AEC) of lung tissue [15]. Our study showed that IL-22R1 mRNA was expressed in the whole murine and human lung tissues, as well as in AEC line A549. However, IL-22 was not detected in fibroblast cell line HFL1 (Figure 4(a)). The phosphorylation of STAT3 was detected rapidly after rIL-22 stimulation and reached the peak around 30 min (Figure 4(b)), corroborating that the A549 cell line is responsive to rIL-22. 

To test whether IL-22 could influence BLM-induced pulmonary damage, BLM was added to epithelial cell cultures in either the presence or absence of rIL-22. The A549 epithelial cells after BLM (100 nU/mL) treatment for over 48 h was shown a significant increase in the expression of mesenchymal markers-*α*-SMA, which is suggestive of the process of EMT. Furthermore, we demonstrated that the addition of exogenous rIL-22 to the culture medium significantly downregulated BLM-induced *α*-SMA in epithelial cells in a dose-dependent manner (Figure 4(c)). Furthermore, analysis of cell viability by CCK-8 revealed that treatment of IL-22 at indicated concentrations could partially reverse the decreased cell viability induced by BLM (Figure 4(d)). Collectively, these results suggest that IL-22 may protect AECs from development towards EMT and impaired viability induced by BLM.

### 3.5. Deterioration of BLM-Induced Pulmonary Fibrosis by Anti-IL-22 Neutralizing Antibody

**Figure 5:**
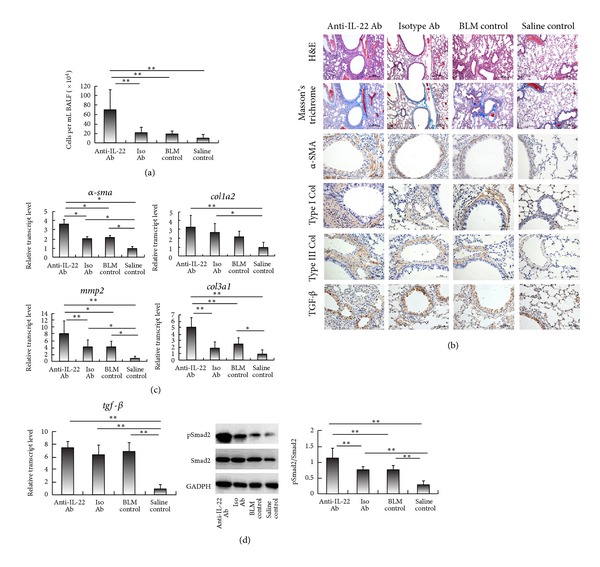
Administration of anti-IL-22 neutralizing antibody enhances BLM-induced pulmonary inflammation and fibrosis. Mice were induced with once bleomycin (BLM) in combination with intraperitoneal injection of anti-IL-22 neutralizing antibody (Anti-IL-22 Ab) or isotype antibody (Isotype Ab) for two consecutive weeks, as compared with BLM-treated mice (BLM control) and saline-treated mice (saline control) without any administration followed. (a) Cell counts of lymphocytes from bronchoalveolar lavage fluid (BALF) were shown. (b) Representative photomicrographs of lung sections were stained with hematoxylin and eosin (H&E) or with Masson's trichrome, as well as by immunohistochemistry for anti-*α*-smooth muscle actin (*α*-SMA), anti-collagen I (Col I), anti-collagen III (Col III), and anti-transforming growth factor (TGF)-*β* antibody. Bars = 200 *μ*m (for H&E and Masson's trichrome staining), or 50 *μ*m (for immunohistochemistry). (c) Levels of transcripts for *α-sma*, *col1a2*, *col3a1*, and *tgf-*β** in the lungs were measured by real-time reverse transcription-polymeras chain reaction (RT-PCR) analysis, normalized to that of saline-treated mice. (d) Level of transcript for *tgf-*β** in the lungs was assessed as described in Figure 4(c) (left). Protein levels of phosphorylated Smad2 (pSmad2), total Smad2, and GADPH in lung homogenates were determined by western blotting (middle). Densitometry was performed, and fold increase in pSmad2 expression after normalization to total Smad2 expression was shown (right). Values in (b), (c), and (d) are the mean and SEM of 5 to 9 mice per group from at least two separate experiments. *P < 0.05; **P < 0.01.

Since substitute rhIL-22 could ameliorate BLM-induced EMT, we suspect that blockage of IL-22 may deteriorate BLM-induced lung fibrosis. To further address our hypothesis, we administrated anti-IL-22 neutralizing antibody (Ab) intraperitoneally daily for 2 consecutive weeks starting from the day of BLM treatment. The levels of IL-22 both in the serum and BALF were decreased significantly confirmed by ELISA (data not shown). Treatment of anti-IL-22 Ab led to even higher lymphocytes in BALF than isotype antibody treated control, suggesting IL-22 may have potential to inhibit the assembly of lymphocytes in BALF induced by BLM (Figure 5(a)). Strikingly, the BLM-induced pulmonary fibrosis was much worse after the IL-22 neutralizing Ab treatment as compared with the isotype Ab-control, shown by H&E and Masson's trichrome-stained lung sections (Figure 5(b)). Immunohistochemical stains of the lung tissues showed an increased expression of Col I and Col III, which was in line with the elevated relative transcript levels of *col1a2* and *col3a1 *measured by real-time RT-PCR (Figures 5(b) and 5(c)). 

EMT markers were also examined after anti-IL-22 neutralizing Ab treatment.

*α*-SMA-expressing myofibroblasts were shown to be increased and mainly distributed peritracheally and perivascularly. Of note, *α*-SMA was also expressed in some epithelial cells, especially in anti-IL-22 neutralizing Ab treated mice. Expression of TGF-*β* examined by immunohistochemistry was higher than that in the isotype Ab-treated lungs (Figure 5(b)). Conversely, neutralizing IL-22 antibodies enhanced BLM-induced transcription levels of *α-sma *and *mmp2 *(Figure 5(c)). The increased expression of transcript for* tgf-*β**by real-time RT-PCR was shown in the anti-IL-22 neutralizing Ab-treated lung tissues as compared with isotype Ab-treated mice, but this did not reach statistical significance (Figure 5(d)). The ratio of pSmad2/total Smad2 was significantly elevated by 147.9% in the anti-IL-22 neutralizing Ab-treated lungs relative to that of isotype Ab-treated control (Figure 5(d)). Taken together, these data provide the evidence that IL-22 regulates the process of BLM-induced EMT and pulmonary fibrosis, likely via TGF-*β*/Smad2 signaling pathway.

## 4. Discussion

Having emerged as an important cytokine in innate immunity, regeneration, and protection from damage, IL-22 plays either a protective or a pathogenic role in different conditions. In the present study, we investigated a BLM-induced pulmonary fibrosis model for 8 weeks and found a progressive process of EMT, aberrant reepithelization, ultimate deposition of ECM, and destruction of lung architectures, accompanied by significantly decreased production of IL-22. Though IL-22 has been reported to have both pathogenic and protective properties depending on the nature of the affected tissue and the local cytokine milieu, here we showed that anti-IL-22 antibody treatment exacerbated the lung fibrosis *in vivo*, indicating a potential protective role of IL-22 in the development of lung fibrosis. Also IL-22 inhibited the overexpression of *α*-SMA and partially reversed the cell viability of epithelial cells induced by BLM *in vitro*, which further confirmed the *in vivo* results. In addition, in order to identify which IL-22 expressed cell subsets play a role in this case, we examined the CD4^+^IL-22^+^, TCR*γδ* ^+^IL-22^+^, NKp46^+^IL-22^+^ cell both in the lung and spleen at the indicated time points by flow cytometry. And we found that IL-22 mainly produced *γδ*T cells were decreased significantly both in the lung and spleen at the 3rd week, indicating TCR*γδ* ^+^IL-22^+^ cell may participate in the regulation of pulmonary fibrosis. Taken together, our studies identified IL-22 as a critical regulator of pulmonary fibrosis after BLM administration, implicating the potential utility of IL-22 in the treatment of pulmonary fibrosis. 

Simonian et al. showed that IL-22-secreting *γδ*T cells could protect lung fibrosis by inhibiting recruitment of *αβ* T cells in a mouse model of Bacillus subtilis-induced hypersensitivity pneumonitis fibrosis [16]. On the other hand, Sonnenberg's group reported a pathogenic role of IL-22 in a model of high-dose-BLM-induced acute lung damage and inflammation. IL-22 has been shown to act synergistically with IL-17A to promote acute pathological airway inflammation. Conversely, anti-IL-22 Ab was found to exacerbate BLM-induced airway inflammation in *il17a* ^−/−^ mice, indicating that IL-22 is tissue protective in the absence of IL-*17*A. Moreover, IL-17A regulated IL-22-mediated protection from BLM-induced airway epithelial cell apoptosis [13]. Braun et al. reported ColV-pretreated animals led to a significant reduction in lung inflammation, which was associated with a significant decrease in the relative expression of interleukin (IL)-6, IL-17, and IL-22 in cells present in BAL fluid at 7 and 14 days after BLM instillation. However, the evidence of lung fibrosis needs to be supplied, and more functional details of IL-22 in the development of BLM induced lung fibrosis need further study [17]. 

Evidence has shown that activated T cells, NK cells, NKT cells, lymphoid tissue inducer (LTi) cells, and LTi-like cells express IL-22, whereas resting or activated B cells, monocytes, monocyte-derived macrophages and immature and mature DCs were not able to produce this cytokine [18–25]. Interestingly, we found that the percentages of IL-22 expressing CD3^+^CD4^−^CD8^−^ oligoclonal *γδ*T cells were significantly decreased both in the lung and spleen at the 3rd week, consistent with the decreased levels of IL-22 as confirmed by western blotting and immunohistochemistry. In the previously reported mouse model of hypersensitivity pneumonitis, the authors found that IL-22-secreting *γδ*T cells could protect from lung fibrosis by diminishing recruitment of CD4^+^T cells to the lung. V*γ*6/V*δ*1^+^ *γδ* T cells are the predominant cell type in the lung producing IL-22 in response to chronic *B. subtilis* exposure. Hence, *γδ*T cells may play the critical role as an inhibitor of pulmonary fibrosis via IL-22 [16]. 

CD3^−^NKp46^+^ cells have been reported to be present in the bone marrow, spleen, thymus, liver, intestines, and lymph nodes and play a role in epithelial cell homeostasis as a distinct subset of NK cell, so-called “NK22” cells which express IL-22 and ROR*γ*T but lack the capacity to perform “classical” NK cell functions such as cytotoxicity and IFN-*γ* production [26]. Our data show that IL-22 expressed CD3^−^NKp46^+^ cells also existed in lung, with no significant difference between BLM-induced lung fibrosis and the saline-treated control. IL-17A expressed NKp46^+^ cells were found neither in the spleen nor in the lung. Thus, our findings are unique in that NK22 cells are also present in the lung, but the function of NK22 cell needs further research.

IL-22 has been shown to bind to the IL-22R1/IL-10R2 receptor complex to mediate its biological effects. Importantly, only the expression of IL-22R1 determines cellular sensitivity towards IL-22 due to the ubiquitous expression of IL-10R2. We demonstrated that IL-22R1 mRNA was expressed in both human and murine lung tissue, and IL-22R1 was expressed only in alveolar epithelial cell line A549 but not in fibroblast cell line HFL1. This result was in agreement with the previous study that IL-22 was found only in primary epithelial cells, but not in alveolar macrophages, monocytes, or neutrophils [15]. Taken together, these data suggest that alveolar epithelial cells may act as the exclusive target cell of IL-22 in the lung. In the next step, we will test the expression of IL-22R1 in the primary cells from the lung tissues of human and mouse. Our data that IL-22 induced the phosphorylation of STAT3 after treating A549 immediately with rhIL-22, reaching the peak around 30 minutes further corroborates this notion. 

TGF-*β* was treated as a central pathogenic contributor in tissue fibrosis. Interestingly, evidence has shown that TGF-*β* could inhibit the IL-22 producing capacity of Th17 cells both in the human [9, 10] and the mouse [11]. Additionally, the development of Th22 cells is downregulated by TGF-*β* [12]. So whether IL-22 could perform the feedback regulation on TGF-*β* signaling deserved our intense investigation. Stimulation of TGF-*β* on the receptor complex led to the activation of Smad2 and Smad3 through direct C-terminal phosphorylation by T*β*RI. TGF-*β*/Smad signaling pathway could mediate fibrosis through the mechanism of EMT. Studies have indicated that lung epithelial cell-specific loss of *α*3 integrin expression reduced EMT and protected from lung fibrosis, apparently by inhibiting tyrosine phosphorylation of *β*-catenin and formation of *β*-catenin/Smad2 complex, and it was confirmed in IPF patients [27]. These findings demonstrated Smad2 was required for the epithelial integrin-dependent profibrotic crosstalk between *β*-catenin and Smad signaling during process of EMT [27]. Additional line of evidence has shown that TGF-*β* could induce A549 cells with an alveolar epithelial type II cell phenotype to undergo EMT, dependent of phosphorylation of Smad2 [5]. Recently Rock et al. proposed that type II alveolar cells (AEC2) are not a major source of myofibroblasts through EMT [3], which is an innovative idea about the origin of myofibroblast. But a lot of humans, mice, and cell data still support EMT as an important event in the development of lung fibrosis [28–33]. So this point still needs further investigation. In our studies, the phosphorylation of Smad2 was found elevated in the lungs of BLM-treated mice. Administration of anti-IL-22 neutralizing antibody resulted in further increase of phosphorylated Smad2, as well as the increment level of TGF-*β*. We think that IL-22 may play a protective role in pulmonary fibrosis through inactivating TGF-*β*/Smad signaling. However, we could not exclude other pathway altered by IL-22 in downregulation of EMT. For example, non-Smad signaling, such as Erk MAP kinases, Rho GTPases, and the PI3 kinase/Akt pathway, could also mediate TGF-*β* induced EMT, and more detailed functional characterization are warranted. 

Pulmonary fibrosis in human can be a fatal disorder characterized by progressive disease that leads to respiratory failure. Our studies revealed a close association between IL-22 and pulmonary fibrosis. Furthermore, we demonstrated that neutralizing IL-22 could lead to the exacerbation of EMT process and an excessive deposition of ultimate collagen. In turn, administration of rIL-22 could inhibit EMT of epithelial cells. Thus, the ability of IL-22 to regulate BLM-induced pulmonary fibrosis both *in vivo* and *in vitro* raises the possibility that IL-22 may act as a potential target to treat diseases characterized by chronic lung inflammation and fibrosis. 

## Abbreviations

Ab: Antibody
AEC: Alveolar epithelial cell
*α*-SMA: *α*-smooth muscle actin
ATCC: American Type Culture Collection
BALF: Bronchoalveolar lavage fluid
BLM: Bleomycin
BSA: Bovine serum albumin
CCK-8: Cell counting kit-8
Col: Collagen
DC: Dendritic cell
ECM: Extracellular matrix
ELISA: Enzyme-linked immunosorbent assay
EMT: Epithelial to mesenchymal transition
FBS: Fetal bovine serum
H&E: Hematoxylin and eosin
h: Hours
IL: Interleukin
ILD: Interstitial lung disease
LTi cell: Lymphoid tissue inducer cell
mRNA: Messenger RNA
Min: Minutes
NK cell: Natural killer cell
OVA: Ovalbumin
PBS: Phosphate buffered saline
RT-PCR: Reverse transcriptase-polymerase chain reaction
TGF-*β*1: Transforming growth factor-*β*1
VILI: Ventilator-induced lung injury.
